# Supplementary material for: High temperature sensitivity of Arctic isoprene emissions explained by sedges
Source: Nat Commun. 2024 Jul 21;15:6144. doi: 10.1038/s41467-024-49960-0 (PMC11271288; doi:10.1038/s41467-024-49960-0)
Supplement: Supplementary file 1 — Supplementary Information [file 41467_2024_49960_MOESM1_ESM.pdf]

Supplemental Information for

High temperature sensitivity of Arctic isoprene emissions explained by sedges

Contents of this file

Supplementary Fig. 1 to Fig. 7

Supplementary Tab. 1 to Tab. 4

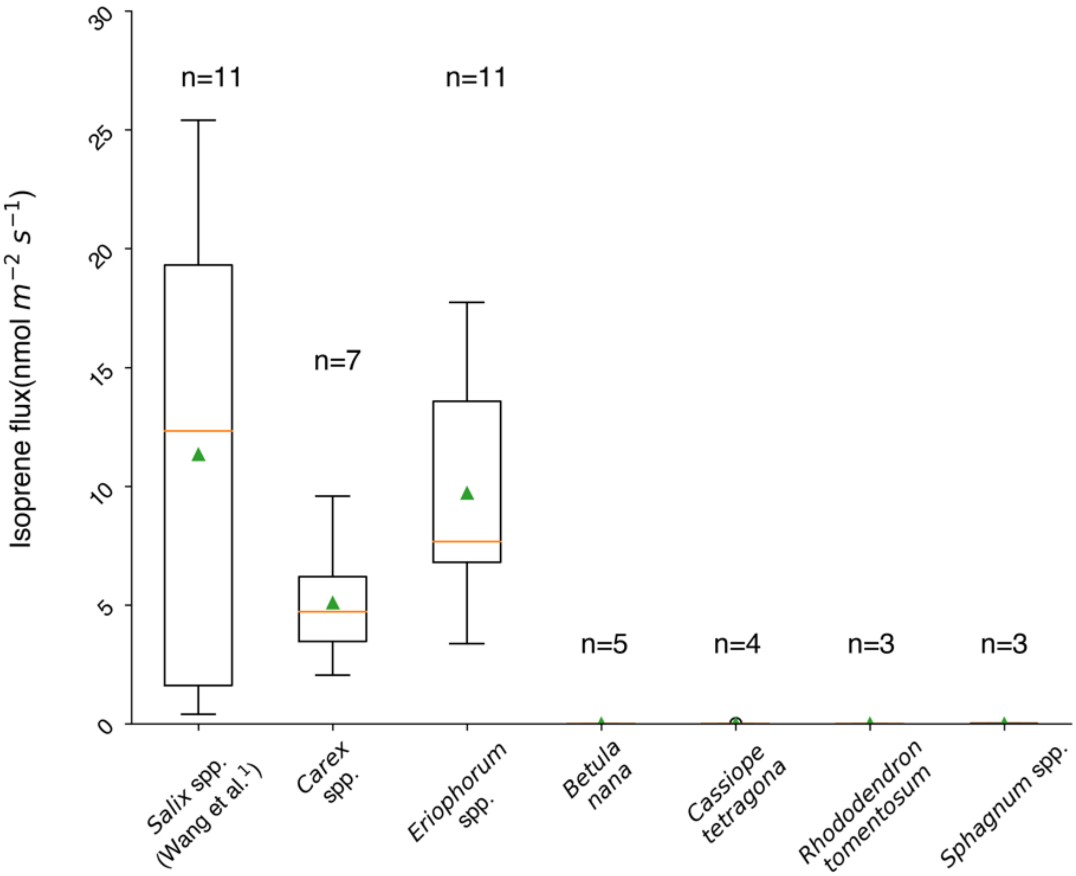

Supplementary Fig. 1. Comparison of leaf-level isoprene emissions from vegetation species at the Toolik Field Station. The measurements were conducted when the leaf temperature was about 30°C under a PPFD of 1000 μmol m^{-2} s^{-1}. The green triangle represents the mean, while the orange line represents the median. The upper and lower boundaries of the box represent the first and third quartiles, respectively. The whiskers extend from the box by 1.5 times the inter-quartile range.

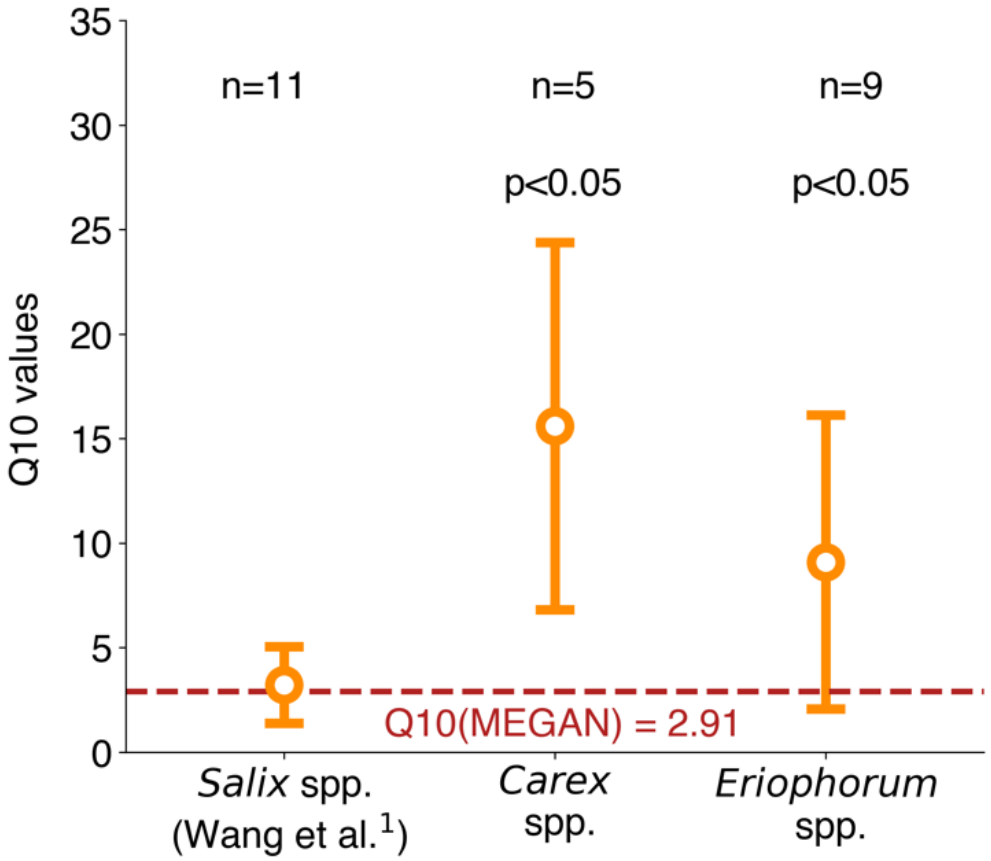

Supplementary Fig. 2. The Q_{10} values between 25 and 35 °C from *Salix* spp. (willows), *Carex* spp., and *Eriophorum* spp. The Q_{10} values between 25 and 35 °C from Arctic sedges are significantly ($p<0.05$) higher than those of willows measured at the Toolik Field Station. The Q_{10} value of MEGAN (=2.91) is presented by a red dashed line. Points and error bars represent mean and standard deviation of Q10.

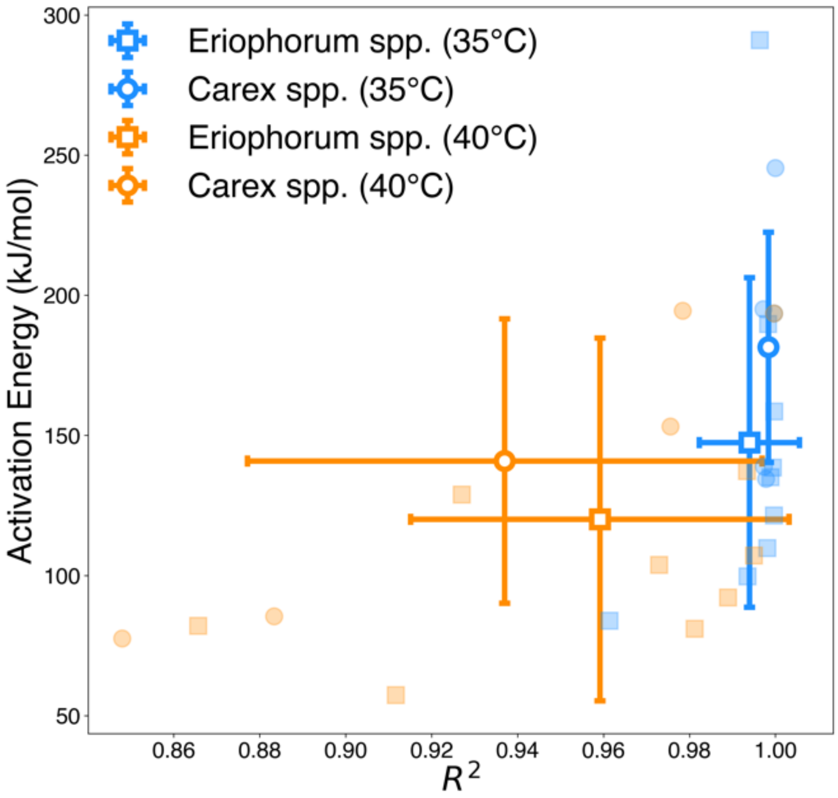

Supplementary Fig. 3. Comparison of temperature sensitivities and R^2 for the fitted curves of sedges from measurements with the highest temperature of 35 °C and 40 °C. Temperature sensitivities are represented by the activation energy in Equation (5). *Eriophorum* spp. and *Carex* spp. are represented by squares and circles, respectively. The colors blue and orange denote the temperature curves fitted with the highest temperatures of 35 °C and 40 °C, respectively. Solid marks indicate the mean values of activation energy and R^2, with error bars representing the standard deviation. Transparent marks represent the values for individual sedges.

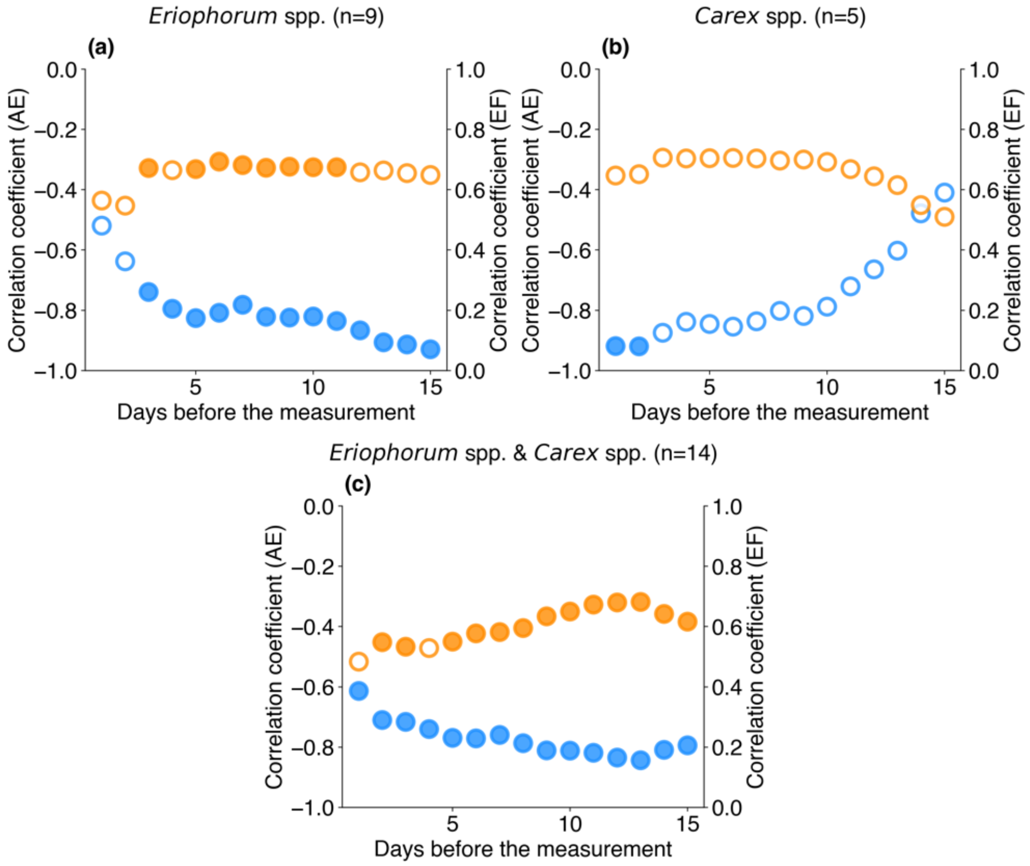

Supplementary Fig. 4. Correlation coefficients of the activation energy (left axis, in blue) and the emission factors (right axis, in orange) of sedges with the mean temperature during the 1 to 15 days preceding the measurement. (a), (b), and (c) display the Pearson correlation coefficients for the activation energy (AE) and emission factors (EF) in relation to the mean temperature of the preceding 1 to 15 days for *Eriophorum* spp., *Carex* spp., and a combined analysis of both species, respectively. Statistically significant correlation coefficients ($p < 0.05$) are indicated by solid filled points.

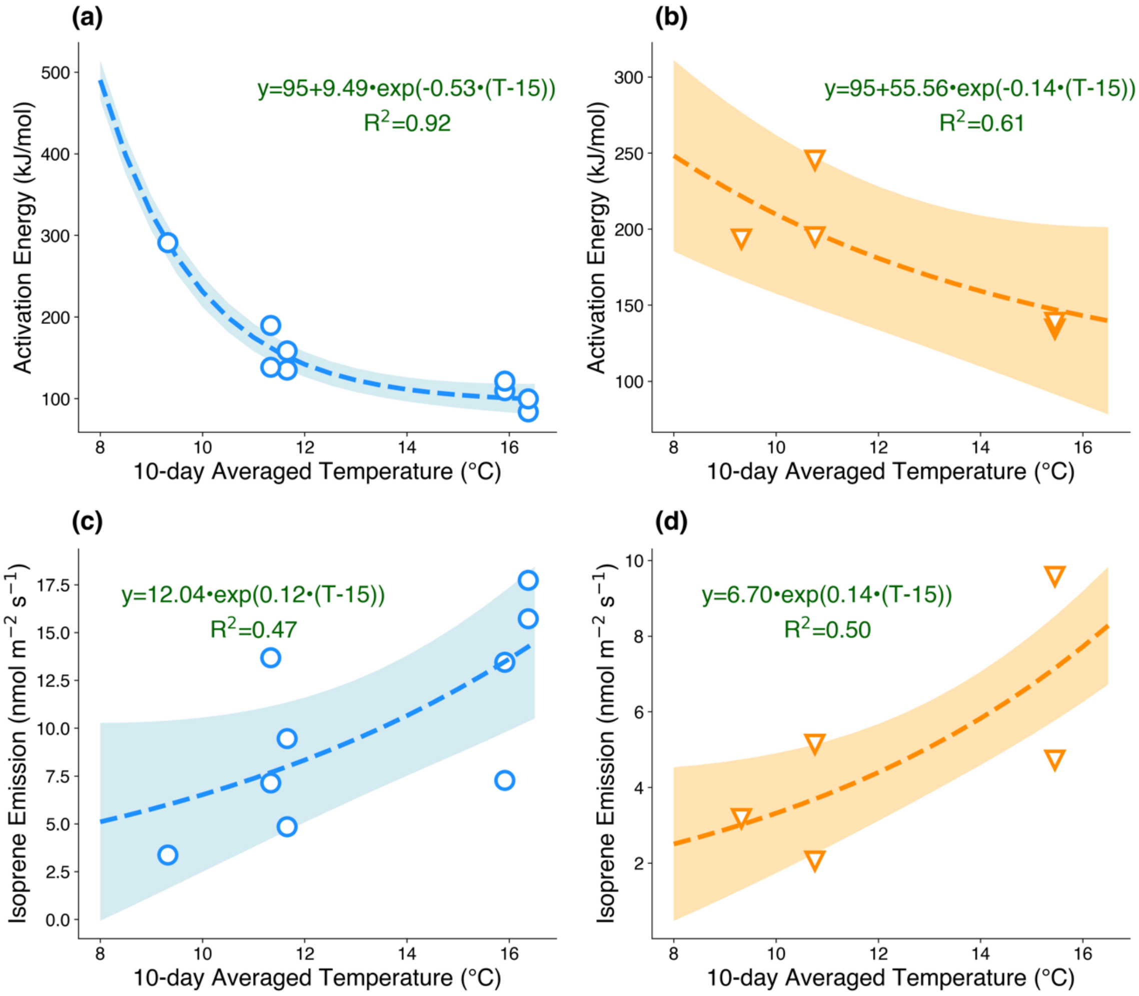

Supplementary Fig. 5. The response curves of the temperature sensitivity and emission factor to the past 10-day average air temperature. (a) and (b) present the relationship between the activation energy or temperature sensitivity to the past 10-day average temperature for *Eriophorum* spp. (blue) and *Carex* spp. (orange). (c) and (d) depict emission factors versus the past 10-day average temperature for *Eriophorum* spp. (blue) and *Carex* spp. (orange). The fitted equation and R^2 are both presented.

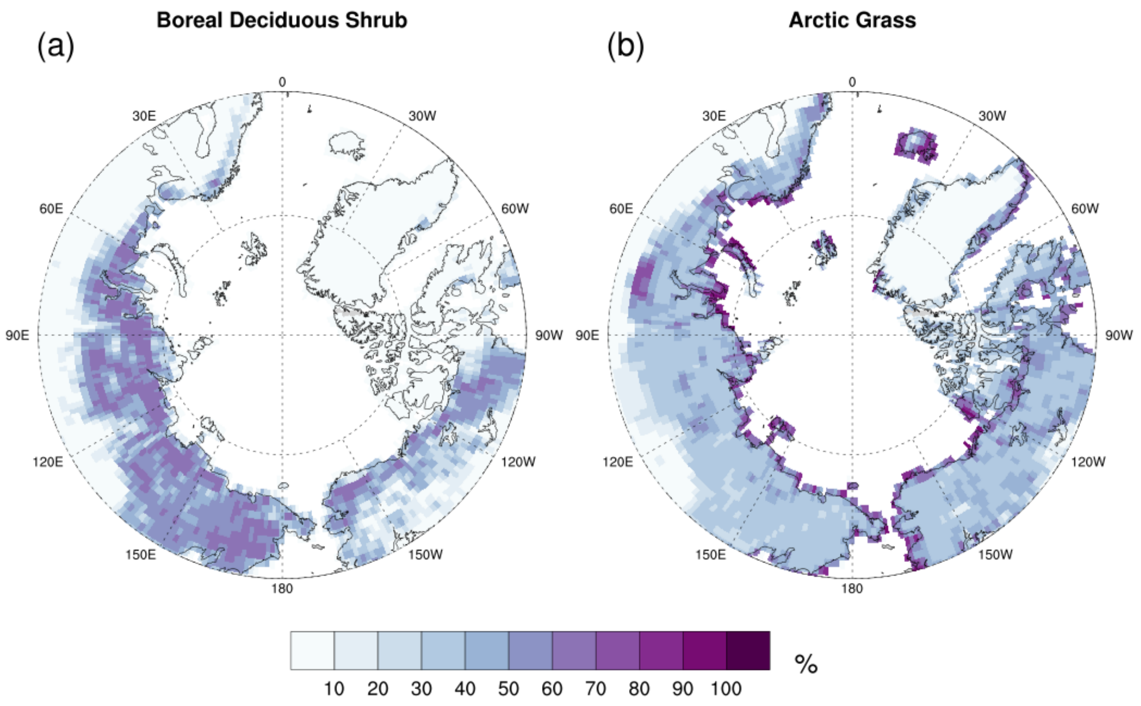

Supplementary Fig. 6. The spatial distribution of cover fraction for the boreal deciduous shrub (a) and Arctic grass (b) in the Community Land Model version 5.

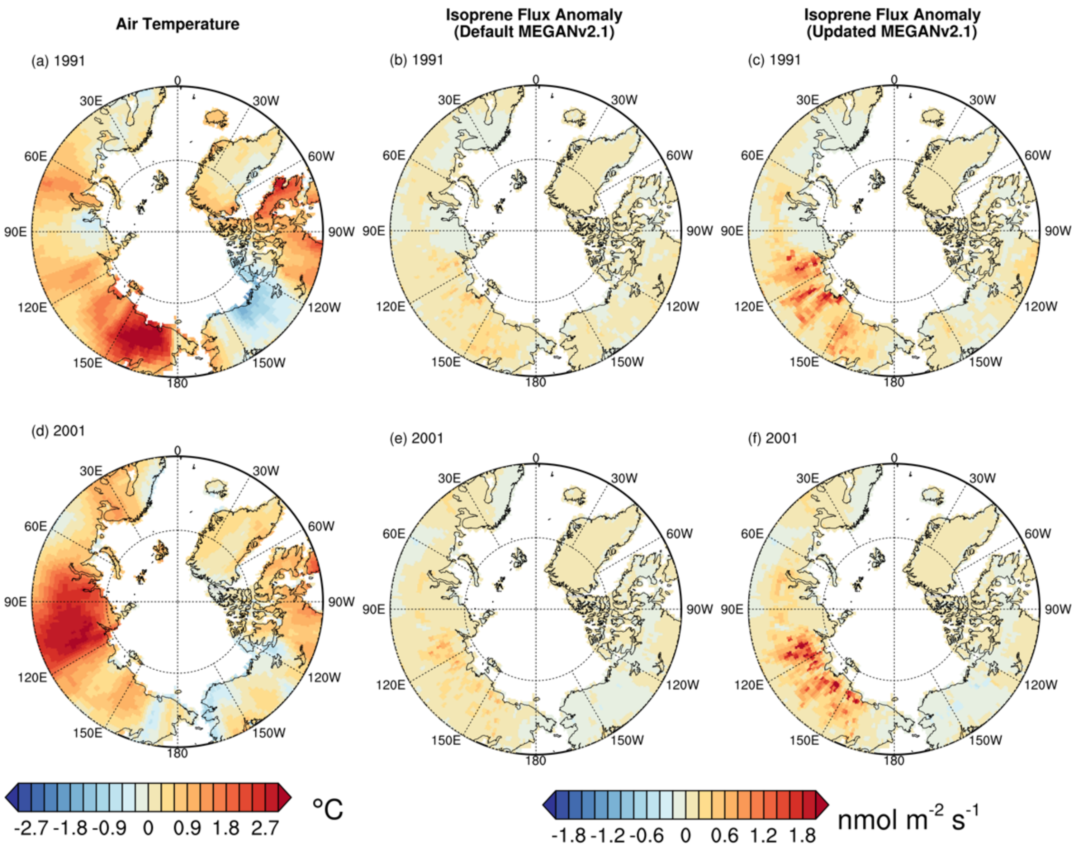

Supplementary Fig. 7. Air temperature and isoprene emission anomalies in the high-latitude regions (north of 60°N) in 1991 and 2001. Air temperature ((a) and (d)) and isoprene emission anomalies in summer estimated by the default and updated MEGANv2.1 in 1991 ((b) and (c)) and 2001 ((e) and (f)).

Supplementary Tab. 1. Details about the flux measurements used in this study.

Site Name	Position	Sample period	Major vegetation species	Reference
Abisko-Stordalen	68.36° N, 19.05° E	Jun. 01 – Oct. 19, 2018	*Empetrum hermaphroditum, Carex rotundata, Betula nana, Rubus chamaemorus, Eriophorum vaginatum, Dicranum elongatum, Sphagnum fuscum, Sphagnum balticum, Drepanucladus schulzei, and Politrichum jensenii*	Seco, et al. [2]
Finse	60.60° N, 7.53 ° E	May 13 – Sep. 26, 2019	*E. hermaphroditum, Salix herbacea and other Salix* spp., *Eriophorum angustifolium, and Carex spp, Ptilidium ciliare and Polytrichum juniperinum, Alectoria ochroleuca, Nephromopsis nivalis, and Cetraria islandica.*	Seco, et al. [2]
Siikaneva	61.83° N, 24.19° E	May 19, - Jun. 28, 2021	*Sphagnum balticum, S. papillosum, S. magellanicum, S. majus, Carex rostrata, C. limosa, C. lasiocarpa, and Eriophorum vaginatum, Andromeda polifolia, Betula nana, Rubus chamaemorus, and Vaccinium oxycoccus.*	Vettikkat, et al. [3]

Supplementary Tab. 2. The performances of models. The statistics of the different temperature response curve models at the Abisko-Stordalen, Finse, and Siikaneva sites with the least square fitting. RMSE and MAE are short for the root mean square error and mean absolute error in the unit of nmol m^{-2} s^{-1}, respectively. T-tests were applied to test the significance between the differences of MAE.

Site	Abisko-Stordalen				Finse				Siikaneva			
-	R^2	Slope	RMSE	*MAE (p<0.05)*	R^2	Slope	RMSE	*MAE (p=0.22)*	R^2	Slope	RMSE	MAE (p<0.01)
Updated MEGAN v2.1	0.81	0.84	0.45	0.24	0.68	0.74	0.58	0.28	0.90	0.89	1.02	0.87
Default MEGAN v2.1	0.78	0.73	0.48	0.21	0.64	0.62	0.60	0.27	0.83	0.80	1.37	0.64

Supplementary Tab. 3. Emission factors of sedges grown near Toolik, AK, USA. Specimens were collected near Imnavait Creek or at Toolik from the local tundra (Toolik). The isoprene emission factor is defined as the isoprene emission rate when the leaf temperature equals 30°C at a photosynthetic photon flux density of 1000 μmol m^{-2} s^{-1}.

Plant ID	Species or Genus	Collection Date	Sample type	Collection Location	Emission Factor (nmol m^{-2} s^{-1})	Experiment
Carex1	*Carex* sp.	Jul. 9, 2022	Leaf	Toolik	3.75	20°C, 30°C
Carex2	*Carex* sp.	Jul. 27, 2022	Leaves	Toolik	3.18	15°C-35°C
Carex3	*Carex* sp.	Jul. 27, 2022	Leaves	Toolik	7.26	15°C-35°C*
Carex4	*Carex* sp.	Jul. 17, 2023	Leaves	Toolik	5.14	20°C-40°C
Carex5	*Carex* sp.	Jul. 17, 2023	Leaves	Toolik	2.06	20°C-40°C
Carex6	*Carex* sp.	Jul. 27, 2023	Leaves	Toolik	4.73	20°C-40°C
Carex7	*Carex* sp.	Jul. 27, 2023	Leaves	Toolik	9.58	20°C-40°C
Eriophorum1	*Eriophorum* sp.	Jul. 16, 2022	Leaves	Imnavait	7.67	20°C, 30°C
Eriophorum2	*Eriophorum* sp.	Jul. 27, 2022	Leaves	Toolik	3.37	15°C-35°C
Eriophorum3	*Eriophorum* sp.	Jul. 27, 2022	Leaves	Toolik	6.48	15°C-35°C*
Eriophorum4	*Eriophorum vaginatum*	Jul. 15, 2023	Leaves	Toolik	4.86	20°C-40°C
Eriophorum5	*Eriophorum vaginatum*	Jul. 15, 2023	Leaves	Toolik	9.46	20°C-40°C
Eriophorum6	*Eriophorum vaginatum*	Jul. 16, 2023	Leaves	Toolik	13.69	20°C-40°C
Eriophorum7	*Eriophorum vaginatum*	Jul. 16, 2023	Leaves	Toolik	7.14	20°C-40°C
Eriophorum8	*Eriophorum vaginatum*	Jul. 30, 2023	Leaves	Toolik	15.73	20°C-40°C
Eriophorum9	*Eriophorum vaginatum*	Jul. 30, 2023	Leaves	Toolik	17.73	20°C-40°C

Eriophorum10	*Eriophorum vaginatum*	Jul. 31, 2023	Leaves	Toolik	13.44	20°C-40°C
Eriophorum11	*Eriophorum vaginatum*	Jul. 31, 2023	Leaves	Toolik	7.28	20°C-40°C

*The VOC samples were only taken at 20 and 30°C.

Supplementary Tab. 4. Emission factors of plants other than sedges at Toolik Field Station. The isoprene emission factor is defined as the isoprene emission rate when the leaf temperature equals 30°C at a photosynthetic photon flux density of 1000 μmol m^{-2} s^{-1}.

Plant ID	Species or Genus	Collection Date	Sample type	Collection Location	Emission Factor (nmol m^{-2} s^{-1})
Willow1	*Salix glauca*	Jul. 13, 2022	Leaf	Toolik	19.53
Willow2	*Salix pulchra*	Jul. 14, 2022	Branch	Toolik	3.84
Willow3	*Salix pulchra*	Jul.16, 2022	Leaf	Toolik	12.33
Willow4	*Salix glauca*	Jul. 25, 2022	Leaf	Toolik	2.07
Willow5	*Salix pulchra*	Aug. 1, 2022	Leaf	Toolik	0.41
Willow6	*Salix glauca*	Aug. 1, 2022	Leaf	Toolik	1.02
Willow7	*Salix pulchra*	Aug. 4, 2022	Leaf	Toolik	1.18
Willow8	*Salix reticulata*	Jul. 25, 2023	Leaf	Toolik	24.8
Willow9	*Salix reticulata*	Jul. 25, 2023	Leaf	Toolik	19.07
Willow10	*Salix reticulata*	Jul. 31, 2023	Leaf	Toolik	15.19
Willow11	*Salix reticulata*	Jul. 31, 2023	Leaf	Toolik	25.39
Birch1	*Betula nana*	Jul. 14, 2022	Branch	Toolik	0.011
Birch2	*Betula nana*	Jul. 16, 2022	Branch	Toolik	0.003
Birch3	*Betula nana*	Jul. 25, 2022	Branch	Toolik	0.008
Birch4	*Betula nana*	Aug. 4, 2022	Branch	Toolik	0*
Birch5	*Betula nana*	Aug. 4, 2022	Branch	Toolik	0.016
Cassiope1	*Cassiope tetragona*	Jul. 17, 2022	Branch	Toolik	0.024
Cassiope2	*Cassiope tetragona*	Jul. 28, 2022	Branch	Toolik	0*
Cassiope3	*Cassiope tetragona*	Aug. 2, 2022	Branch	Toolik	0*
Cassiope4	*Cassiope tetragona*	Aug. 6, 2022	Branch	Toolik	0*
Rhododendron1	*Rhododendron tomentosum*	Jul. 17, 2022	Branch	Toolik	0*
Rhododendron2	*Rhododendron tomentosum*	Jul. 28, 2022	Branch	Toolik	0*
Rhododendron3	*Rhododendron tomentosum*	Aug. 2, 2022	Branch	Toolik	0*
Sphagnum1	*Sphagnum sp.*	Jul. 14, 2022	Leaves	Toolik	0.001
Sphagnum2	*Sphagnum sp.*	Jul. 21, 2022	Leaves	Toolik	0.03
Sphagnum3	*Sphagnum sp.*	Jul. 21, 2022	Leaves	Toolik	0.011

* The measurements are lower than the blank tube concentration.

Reference

1 Wang, H. *et al.* Arctic Heatwaves Could Significantly Influence the Isoprene Emissions From Shrubs. *Geophysical Research Letters* **51**, e2023GL107599 (2024).

2 Seco, R. *et al.* Strong isoprene emission response to temperature in tundra vegetation. *Proceedings of the National Academy of Sciences* **119**, e2118014119 (2022).

3 Vettikkat, L. *et al.* High emission rates and strong temperature response make boreal wetlands a large source of isoprene and terpenes. *Atmos. Chem. Phys.* **23**, 2683-2698 (2023).
